# Climate Change and Zoonotic Disease Outbreaks: Emerging Evidence from Epidemiology and Toxicology

**DOI:** 10.3390/ijerph22060883

**Published:** 2025-05-31

**Authors:** Abdallah Borham, Kadria Abdel Motaal, Nour ElSersawy, Yassmin F. Ahmed, Shuaib Mahmoud, Abobaker Salem Musaibah, Anwar Abdelnaser

**Affiliations:** Institute of Global Health and Human Ecology, School of Sciences and Engineering, The American University in Cairo, P.O. Box 74, New Cairo 11835, Egypt; abdallahborham@aucegypt.edu (A.B.); kkmotaal@aucegypt.edu (K.A.M.); nourhamdy@aucegypt.edu (N.E.); yassmin.f.ahmed@aucegypt.edu (Y.F.A.); mahmoudshuaib@aucegypt.edu (S.M.); abobaker@aucegypt.edu (A.S.M.)

**Keywords:** climate change, zoonotic diseases, emerging infectious diseases, disease vectors, One Health, triple planetary crisis

## Abstract

Background: Disruptions in the mesh of the ecosystem come with implications that severely harm the sustainability and the equilibrium of life. Interactions of humans, animals, and many other organisms, along with the whole ecological complex, have given birth to zoonotic diseases, which can vary in type and burden. Collaborative efforts put into the prioritization of environmental, animal, and human health are envisioned as “One Health”. Understanding vector ecology and the varying mechanistic ways of transmission is crucial for constructing effective One Health surveillance tools and warning systems. Methods: We identified the literature available concerning the subject matter. We utilized scholarly databases to gather research for the last 10 years using predefined keywords. Objectives: This review aims to synthesize current knowledge on the interconnection between climate discrepancies, ecological alarms, and the emergence and spread of zoonotic diseases. We attempted to provide recommendations for future research and policy interventions. Results: Human activities have significantly impacted disease-carrying vectors and wildlife habitats, aiding their proliferation and the spillover of diseases. Global frameworks incorporating One Health principles enhance global preparedness for future health threats. Applying the integrated One Health Surveillance has strengthened early warning systems. Interdisciplinary collaborations and tools like OH-EpiCap, a comprehensive tool that assesses and enhances the capacities of One Health surveillance systems, have significantly contributed to responding to infectious disease outbreaks, as seen in the Netherlands, reducing the risk of tick-borne diseases. Conclusions: Strides have been made with comprehensive processes that identify and prioritize zoonotic diseases of most significant concern and burden, such as OHZDP, approaches like One Health, and other theories considered. A proactive and integrated approach will build resilience against potential outbreaks and ensure a healthier future for our planet and its inhabitants.

## 1. Introduction

**Figure 1:**
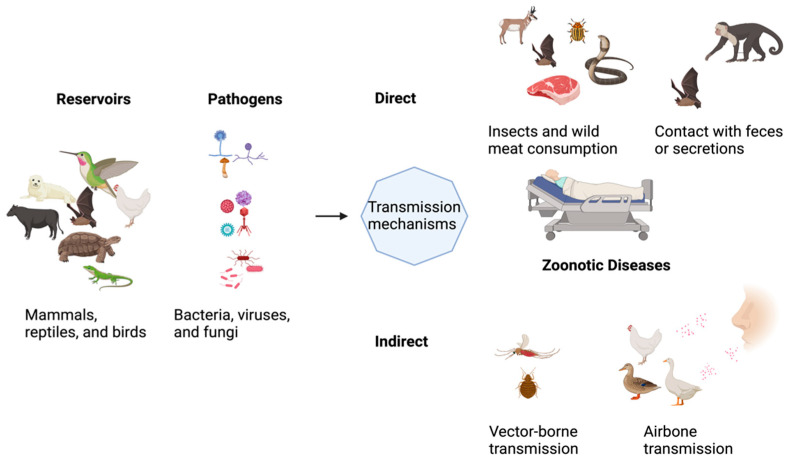
Illustration of the concept of pathogen transmission mechanisms.

Ecosystem, Climate, and all living forms are interlinked in endless ways. In Greek, “Zoon” means animal, while “noses” implies illness. According to the World Health Organization (WHO), any disease or infection naturally transmissible from vertebrate animals to humans or humans to animals is classified as zoonosis [1]. Diseases transmitted from animals to humans and vice versa through direct or indirect means are called Zoonoses [2,3]. Evidence suggests that animals are key players in most infectious diseases affecting humans. The contribution of zoonoses in human infections could exceed 60%, with 70% of these coming from wildlife animal species only. Those animals could be pets, companion animals, and birds. Fish and aquatic animals also contribute to the emergence of infectious diseases of zoonotic origin. Insects are one of the zoonotic disease vectors and act as an intermediate reservoir for various pathogens. They have high nutritional benefits and are heavily relied on as food sources in Asia, Africa, and South America, intensifying the risk of spreading zoonotic disease. With nearly 2 billion people worldwide considering beetles, caterpillars, insects, and many other insects as favorable food sources, it is clear how potential zoonoses could be transmitted to these populations. That said, edible insects increase the risk of zoonoses carrying various bacterial, parasitic, and foodborne pathogens. Figure 1 shows that zoonotic diseases have varying etiologies and are caused by multiple pathogens. Zoonoses can be classified according to the etiological agents, transmission cycle (maintenance cycle), mode, and reservoir hosts [4].

In terms of the causing agent, Zoonoses are classified into bacterial, viral, parasitic, fungal, rickettsial, chlamydial, mycoplasma, protozoal, and acellular non-viral pathogenic agents. Based on etiology, bacterial pathogens are the cause of most of the zoonotic diseases. The reservoir host can be a wildlife or non-wildlife animal (domestic), categorizing the zoonotic disease into three types that could be transmitted from animal to human, human to animal, or bi-directional developing diseases in the two hosts. The literature has shown that 60.3% of emerging infectious diseases (EIDs) were of zoonotic origin. That said, 71.8% of these EIDs of zoonotic origin were of wildlife roots. This entails that the non-wildlife-originated pathogens constitute 28.2% of all zoonotic pathogens. Although the exact stratification of non-wildlife was not clearly stated, 45 bovine zoonotic pathogens were identified. The largest taxonomic group in these bovine pathogens is bacteria, constituting 42% [5,6].

Earth’s unique climate has undergone severe remodeling in the past few hundred years. Earth has undergone eight cycles of glacial morphoses and modifications in the last 800,000 years before the modern era. Since the last ice age 11,700 years ago, the contemporary climate era began with the commencement of human civilization. Moreover, the rate of climate change since the 19th century has accelerated in a way that has never been witnessed before. Atmospheric carbon dioxide has exceeded the prior levels. Human interference and interaction with Earth have led to unwarranted trends in climate change and global warming [7,8,9]. Tracing and monitoring of human activities, zoonotic transmission cycles, climate shifts, and environmental alarms are instrumental in preparing for future zoonotic threats [10].

Furthermore, investigating the impacts of climate change on the global zoonotic disease transmission trends will allow for rapid and steadfast interventions. The climate outline is affected by various components such as land and ocean temperatures, sea level, seawater acidity, extreme weather events, wind patterns, and other land/soil characteristics. The anthropogenic emission of greenhouse gasses (GHGs) is a key determinant of the deteriorating climate. The Earth’s global mean surface temperature has increased by 1.09 °C compared to the pre-industrial era, which is substantial [8]. Various factors influence the likelihood of extreme weather disasters. The interactions of the Earth’s atmosphere, the other spheres, and the hydrological cycle can create hazardous events for the entire human populace. The El Nino-Southern Oscillation (ENSO), a dysregulated periodic alteration in the surface temperature and atmospheric pressure of the equatorial Pacific Ocean, consists of two phases: the warming (El Nino) and cooling phase (La Nina); along with other oscillations, they collectively contribute to drastic weather events [11]. Geoclimatic changes and global warming have led to the persistence of zoonotic diseases, expanding their epidemiological limits.

With that in mind, the dynamics of the hosts, vectors, and pathogens have been entirely remodeled, disrupting the natural interconnection between them. This disturbance could induce multiple hazards for all the factors inside the ecosystem. Malaria, cholera, plague, and dengue, among many emergent zoonotic diseases, were seen to be aligned with ENSO-related intense weather events [12]. Strong associations were observed, and the results have shown how climate affects zoonoses activity enormously. Vector-borne, mosquito-borne, Sand fly-borne, Tick-borne, waterborne, foodborne, airborne, and rodent-borne zoonoses are the culmination of the climate-driven zoonotic projection [8]. Almost all human systems and organs are affected, such as the heart, brain, and gut, which are all at severe risk of deterioration and disease. As a result, mortality and morbidity have skyrocketed [13].

Extreme temperatures, cold and hot, have deleterious effects on the cardiovascular system. The natural cooling mechanisms of the human body, as a reaction to extremely high temperatures, can lead to exhaustion of the heart and lungs. Heat fainting and strokes are inevitable in heat waves, with death as a considerable possible event. Also, heat weather can increase ozone levels, leading to severe acute and chronic respiratory complications resulting from airway inflammation and lung structural damage. Unsettled weather variations disrupt endocrine homeostasis. This fluctuation in the temperature activates the sympathetic nervous system aggressively. The stress mode obliges the body to secrete large amounts of epinephrine, or “adrenaline”, causing an alerting response to all body organs, prominently causing hypertension and tachycardia. The pituitary gland, as one of the master glands in the body, becomes anxious and unleashes its gamut of hormones to the bloodstream, causing multiple dysfunctions in normal physiologic processes and eventually turning into a disease. Enormous pathologic effects could arise from discrepancies in the temperature as a result of climate change. Aggravation in non-communicable diseases (NCDs), maternal and child health, communicable diseases, and many other disease burdens may befall [14,15].

Zoonoses pose a tremendous Global Public Health threat. Not only are humans affected but also animals: both livestock and wildlife [16,17]. Consequently, deleterious challenges befall our environment, distressing our climate to be metamorphosed. Death, poverty, pandemics, catastrophic expenditures, environmental injuries, and famine have prevailed in this Holocene [17]. Annually, it is estimated that 2.7 million individuals die of zoonoses, not to mention the countless cases of illnesses with varying severities [5]. A good example of a lethal pandemic that arose from a suspected bat–human interaction is that that gave birth to SARS-CoV-2 back in 2019 [8]. Food insecurity, among other concerns, could originate from animal–human diseases.

EIDs may evolve because of the evolution of organisms that differ in habitat and ways of transmission [18]. Accordingly, antimicrobial resistance and ecological disfigurement play a part in the emergence and re-emergence of EIDs brought about by zoonotic pathogens [2]. Niche paradigms of action and strategy are crucial to prevent, mitigate, and fight the spreading of zoonoses. Utilizing the One Health paradigm could be paramount in combating such threats. The One Health approach has an ancient nature of continuously extrapolating veterinary knowledge and inferring it into human medicine [3]. One Health is a holistic concept that aims to include all possible components that could lead to or aggravate the occurrence of disease into the equation of control and prevention. Incorporating environmental, ecological, animal, and human factors instead of fragments is the true power of such a concept, supporting the prevention and mitigation of zoonotic diseases. The cooperation, collaboration, and coordination of various stakeholders and sectors are fundamental to implementing the One Health approach in real-world settings. This stance could be seen in inter-governmental collaborations, international alliances, global commissions, national task forces, and many other forms of multi-sectoral and integrated coherences that unify efforts in the face of malignant diseases and outbreaks [6,7]. This review aimed to highlight the most recent steps taken in the quest against zoonoses and climate damage. We synthesized the current knowledge available regarding zoonoses and climate change. We attempted to find the gaps and fill them with a practical/pragmatic level of scrutiny and evaluation, predicting what lies ahead while presenting what has already been achieved. We set forth valuable recommendations for policy reforms and systems adjustment. We brought instrumental insights to foster productive discussions and practical actions toward controlling zoonoses’ emergence and spread.

## 2. Methods

This study was conducted as a narrative review rather than a systematic review. The process was collaborative, with different sections assigned to multiple authors based on their areas of expertise. Each author conducted an independent review of the assigned literature, and the final manuscript was collectively evaluated to ensure consistency, coherence, and comprehensive coverage of the topic. Discrepancies or differences in interpretation were resolved through discussion and consensus among all authors before finalizing the review.

A search strategy was applied to identify the relevant literature that tackled the symbiotic interrelation and interconnected relationship between climate change and zoonotic disease outbreaks. Predefined eligibility criteria were used. The inclusion criteria were the following: (1) focus on Climate Change and Zoonotic Diseases; (2) publication in Peer-Reviewed Journals or authoritative public health reports; (3) studies published in the last 15 years; and (4) articles in the English language only. The exclusion criteria were the following: (1) irrelevant articles; (2) non-peer-reviewed Sources; and (3) articles in languages other than English. Three hundred articles were initially retrieved from PubMed, Google Scholar, Scopus, and Science Web databases. Also, reports from International Organizations were included to cover this policy-related topic from non-scholarly perspectives. Concepts and keywords related to the research topic were used, such as climate change and zoonosis, global warming, One Health, zoonotic diseases, mitigation and adaptation, and strategies for One Health. After removing duplicates and conducting title and abstract screening, 182 articles were excluded due to irrelevance, methodological limitations, or lack of required data. The remaining 126 articles were thoroughly reviewed and included in the final analysis.

## 3. One Health Framework

The COVID-19 pandemic, caused by SARS-CoV-2, highlighted the interconnected nature of global health threats, which often involve multiple socio-economic, cultural, and environmental factors beyond a single biological agent. This complexity results in “syndemic” effects, where adverse health outcomes are intensified by interactions with other harmful conditions [19]. To date, health systems have primarily operated in silos, limiting comprehensive global preparedness. To be effective, preparedness must proactively anticipate and mitigate risks by considering various factors beyond biological agents. Most emerging pathogens have zoonotic origins [20]. The One Health approach is a collaborative integrated approach recognizing human, animal, and environmental health interconnection. It aims to prevent and mitigate health threats by fostering cross-sectoral cooperation and sustainable solutions. It highlights the necessity of considering the environmental drivers behind zoonotic diseases. Climate change exacerbates conditions that foster zoonotic disease outbreaks, including deforestation, changes in vector populations, increased human–animal interactions, environmental exploitation, and socio-economic factors. However, integrating One Health into governmental and international plans faces challenges due to fragmented governance, lack of cross-disciplinary collaboration, and funding [19]. Rather than isolated national strategies, there is an urgent need for global frameworks that incorporate One Health principles into broader policies to enhance global preparedness for future health threats.

Although the concept of One Health is not new, it has evolved significantly over time. Its origins can be traced from Aristotle’s work to the modern idea of Zoobiquity, coined by Natterson and Bowers in 2012. It highlights the interconnection between ecosystems, animals, and humans [21]. The definition of One Health is complex due to its multidisciplinary scope, which includes a range of scientific fields. In the 21st century, it represents a reimagined approach to health management, driven by rapid environmental changes primarily resulting from exponential population growth and urbanization over the past century. In recent years, numerous global calls have advocated adopting the One Health approach, which has become increasingly integrated into national pandemic response strategies [22].

In response to this growing momentum, the One Health High-Level Expert Panel (OHHLEP) was established in May 2021 to provide advisory support to the concerned Quadripartite organizations: the Food and Agriculture Organization (FAO), the World Organization for Animal Health (WOAH), the United Nations Environment Program (UNEP), and the World Health Organization (WHO). The panel delivers evidence-based scientific insights and guidance on One Health issues [23]. The panel analyzes potential health crises that emerge from the interaction between humans, animals, and ecosystems, identifies research gaps, and formulates strategic approaches to reduce the likelihood of zoonotic pandemics. This strategy fosters coordination and institutionalization of the One Health approach, particularly in areas that heighten pandemic risks. It also aimed to develop a comprehensive monitoring and early warning system.

To ensure the sustainability and effectiveness of their efforts, OHHLEP introduced a Theory of Change (ToC), which functions as a guiding framework not only for the panel but also for the Quadripartite organizations and other groups working on similar goals. The Theory of Change (ToC) is a strategic planning tool that outlines how and why a particular initiative or program will achieve its desired outcomes. It helps organizations map out the steps required to achieve long-term change by breaking down complex goals into manageable actionable steps [23]. This framework is designed to evolve as a new evidence-based science, allowing for ongoing review and adjustments to fit the shifting global landscape of socio-political and institutional realities [24]. OHHLEP worked on creating consensus on a cohesive definition of One Health, facilitating its translation into actionable policies to support a unified understanding among stakeholders globally. The Definition proposed by Adisasmito et al. [24] states the following:

*One Health is an integrated, unifying approach that aims to sustainably balance and optimize the health of people, animals, and ecosystems. It recognizes the health of humans, domestic and wild animals, plants, and the wider environment (including ecosystems) as closely linked and interdependent. The approach mobilizes multiple sectors, disciplines, and communities at varying levels of society to work together to foster well-being and tackle threats to health and ecosystems while addressing the collective need for healthy food, water, energy, and air, acting on climate change, and contributing to sustainable development.*

*(p. 11) [24]*

### 3.1. The 4 Cs Concepts in the One Health Approach

**Figure 2:**
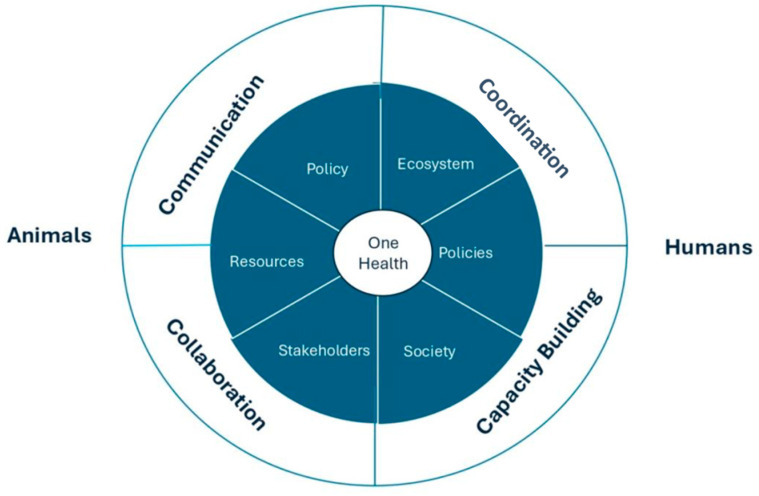
A Diagram illustrating the One Health concepts.

The “4 Cs” are key concepts to enhance global health security and ensure the success of the One Health Approach in addressing health risks where human, animal, and environmental health intersect, as shown in Figure 2. The first concept, communication, focuses on the transparent exchange of information among all concerned stakeholders from the healthcare sector, policymakers, and community members. The second concept, coordination, points to harmonizing the work of all involved parties toward a common goal to respond to multifaceted health issues consistently. The third concept, collaboration, advocates for comprehensive, interdisciplinary, and multisectoral partnerships to create strategies addressing the interrelated nature of human, animal, and environmental health. The fourth concept, capacity building, is concerned with building the aptitudes, supplies, and infrastructure required to apply One Health initiatives successfully. This includes training across disciplines, improving health systems, and providing the tools needed for a long-term sustainable One Health response [23]. The key principles of One Health are the following:

1. Equity across sectors and disciplines to ensure fairness and equal treatment among various fields and areas of study;
2. Sociopolitical and multicultural parity to recognize that all individuals have the same rights and opportunities and prioritize the inclusion and active participation of all communities, particularly marginalized ones;
3. Socio-ecological balance to foster a harmonious relationship between humans, animals, and the natural environment; acknowledging the importance of biodiversity; ensuring equitable access to natural spaces and resources; and recognizing the intrinsic value of all living organisms within ecosystems;
4. Stewardship emphasizes the human responsibility to adopt sustainable practices and behaviors that uphold animal welfare and the integrity of ecosystems, thus safeguarding the health and prosperity of both current and future generations;
5. Transdisciplinarity and multisectoral collaboration encourage cooperation among all relevant fields to integrate modern and traditional knowledge and ensure a broad representation of different perspectives.

### 3.2. One Health Strategies

**Figure 3:**
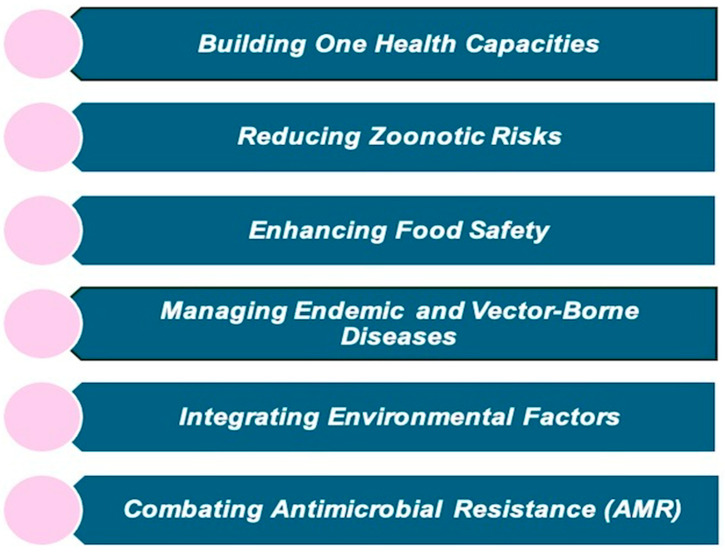
Six action tracks of OHJPA.

The One Health High-Level Expert Panel (OHHLEP) developed the One Health Joint Plan of Action (OHJPA) to tackle health challenges arising from the interaction between human, animal, plant, and environmental health to ensure the effective implementation of the One Health framework. This plan underscores the commitment of the founding organizations, “FAO, WHO, WOAH, and UNEP”, to support existing One Health efforts and enhance global, regional, and national health systems. It aims to improve the ability to prevent, predict, detect, and respond to health threats while supporting sustainable development [25]. Policy advice, technical assistance, and support help countries set national targets, prioritize health goals, and develop relevant legislation and programs. Additionally, the framework focuses on reviewing and identifying overlaps in existing One Health initiatives to ensure better coordination, avoid redundancy, and improve efficiency. It provides capacity-building, cross-sectional, and collaborative approaches that allow for optimum resource allocation and distribution that integrate all sectors, disciplines, and stakeholders for long-term sustainability and effectively address health risks. The OHJPA is structured around six interconnected action tracks to improve health outcomes and sustainable ecosystem management, as shown in Figure 3 [26].

Each action track includes specific activities, deliverables, and timelines to achieve key objectives such as promoting multi-sectoral health, reducing pandemic risks, supporting community-based disease control, enhancing food safety, and preserving biodiversity and ecosystems. In addition to its specific goals, the OH JPA incorporates multidisciplinary principles such as systems thinking, advocacy, public-private partnerships, and effective governance, which apply across various sectors to enhance collaboration and impact. It also prioritizes the inclusion of traditional knowledge from local and Indigenous communities. These principles help integrate the six action tracks, ensuring they work together to holistically address everyday health and environmental management challenges.

### 3.3. Challenges and Barriers to the Implementation of the One Health Framework

Several barriers hinder the effective implementation of One Health interventions. According to Yopa et al., they can be classified under three main areas, as follows [27]:

- Political and Institutional Barriers

Weak governance and leadership were significant barriers to One Health interventions, especially in developing countries. Local governments have struggled to coordinate actions among stakeholders and mobilize the necessary resources for effective implementation. There is resistance from both national and international bodies to adopt a unified approach fully;

- Operational and Logistical Challenges

Several operational issues hinder the implementation of One Health strategies. Adequate planning and clear guidance for stakeholders are often missing, complicating objectives setting and measurement. Insufficient financial and human resources and the lack of specific training and practical experience among stakeholders lead to delays and interruptions. Furthermore, ineffective collaborations result in the duplication of efforts, while inadequate disease surveillance systems hinder early detection and rapid response to health threats;

- Cultural and Communication Barriers

Communication between stakeholders, especially human and animal health professionals, was limited, causing difficulties in coordinating disease surveillance and developing joint action plans. Additionally, the insufficient involvement of local communities in planning and implementation reduces their engagement and acceptance, resulting in poor communication and awareness of the importance of integrating human, animal, and environmental health into One Health interventions.

## 4. Classification of Zoonoses

**Figure 4:**
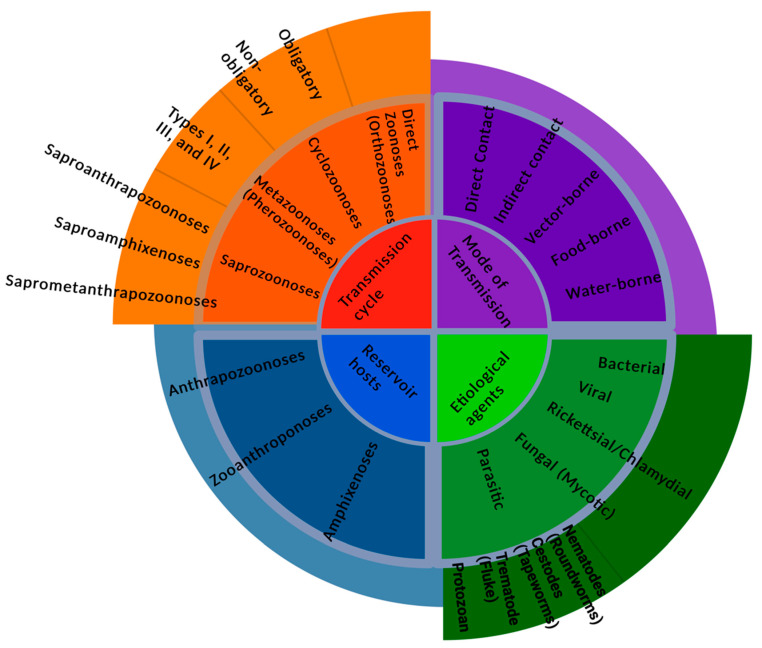
An abstract showing the various ways of classifying zoonoses.

Based on etiological agents, zoonotic diseases can be bacterial, viral, Rickettsial or Chlamydial, Mycotic or Fungal, and parasitic, as shown in Figure 4 [5]. However, based on the transmission cycle, which is considered the old classification, zoonotic diseases can be in the form of direct zoonoses (Orthozoonoses), which need single vertebrate species for their maintenance [4,28]. Cyclozoonoses require two or more vertebrate hosts to complete the transmission cycle of an infectious agent. Metazoonoses (Pherozoonoses) require both vertebrate and invertebrate species. Invertebrate hosts, which are infectious agents, may multiply, develop, or remain dormant. This type of zoonotic transmission is subdivided into four subtypes according to the number of vertebrate and invertebrate hosts [4]. Saprozoonosis requires a non-animate substance to complete the life cycle in addition to a vertebrate or invertebrate host. An infectious agent may multiply, develop, or propagate in an inanimate site. It is subdivided into three subtypes: it can be transmitted via non-animated substances as in Saproanthrapozoonosis; it can be equally shared in nature by humans and animals and be transmitted through inanimate objects as in Saproamphixenosis; or it could require vertebrate hosts, invertebrate hosts, and inanimate objects for the completion of the transmission cycle known as Saprometanthrapozoonoses [4].

Zoonotic diseases can be categorized based on reservoir hosts [5]. Anthrapozoonosis is developed in domestic and wild animals that are found in nature. Human beings can become infected by animals from unusual circumstances such as occupational contact or unusual ways of food consumption. Zooanthroponosis is a disease that usually passes from humans to other vertebrate animals. Furthermore, Amphixenosis is a disease in which the causative agent can pass from human to animal and vice versa [4].

Routes of transmission can determine the type of zoonosis. It can be by direct contact with an infected animal’s saliva, blood, urine, mucous, droplets, feces, or other body fluids [29]. Vector-borne is indirect contact, such as being bitten by a tick or an infected insect like a mosquito or a flea [30]. Foodborne transmission mode is ingesting food contaminated with the causative agents from feces or other body fluids excreted by an infected animal. As a result, contaminated food can cause illnesses in humans and animals. Waterborne transmission routes are due to drinking contaminated water, which carries pathogens from the feces of an infected animal [31].

## 5. Zoonotic Disease Dynamics and Climate Change

### 5.1. Changes in Vector Ecology

**Figure 5:**
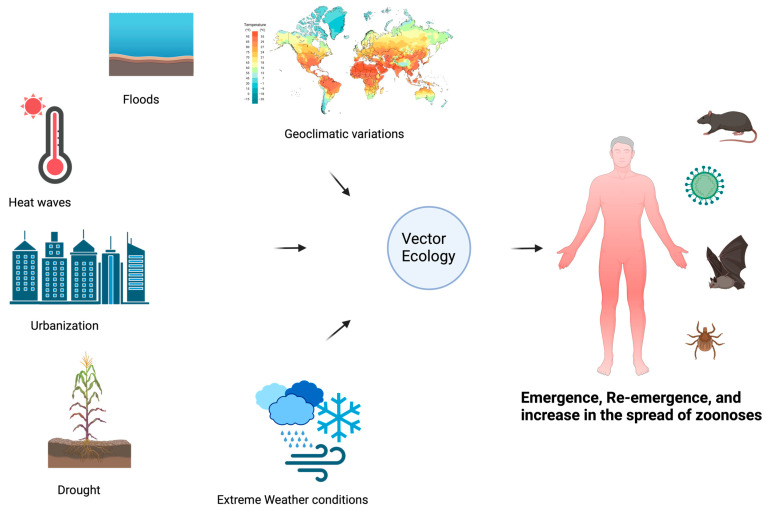
An illustration of the dynamics of vector ecology and how this affects the emergence, re-emergence, and projection of zoonotic diseases.

Several transformations are happening in the human environment, including climate change, changes in human demographics, behavior, land use practices, and environmental changes at both large and small scales. These changes, alone and in combination, alter the relationship between humans and infectious disease agents, leading to the apparent emergence of contagious and zoonotic diseases [32], as shown in Figure 5. These changes also influence the vectors (e.g., ticks, fleas, black flies, mosquitoes, and sand flies) of zoonotic parasites and change their relationships with humans [33]. Due to their ectothermic nature, arthropod vectors are particularly vulnerable to changes in climatic conditions, such as alterations in rainfall and humidity. External environmental conditions influence their internal temperature and biological functions [34].

The increasing temperatures and unpredictable precipitation patterns associated with climate change have created favorable conditions for the proliferation of disease-carrying vectors such as ticks and mosquitoes and species diversity, which induces physiological changes and affects the biting rates of sand flies, their diapause, and the development of pathogens (protozoa) within the vector [8,35]. The spread and proliferation of *Aedes aegypti* mosquitoes, vectors of Dengue; Yellow fever; Chikungunya; and Zika viruses, pose an enormous risk to unexposed populations and may lead to new epidemic outbreaks [36]. Various types of mosquitoes are also vectors for diseases such as West Nile fever, Malaria, Japanese encephalitis, and Lymphatic filariasis, which are all highly propagating and rapidly spreading diseases affected by the varying environmental conditions [37]. With that, humans adjust to climate change by modifying their environments. For instance, drought conditions have led communities in southeastern Australia to implement water tanks, which are anticipated to increase the spread of *A. aegypti* and heighten the risk of emerging and re-emerging diseases in those regions [38].

Most vectors need stagnant and standing water sources for reproduction. This habitat strongly impacts their reproduction, as their larvae develop in and on the water where their eggs are laid, and all the development occurs there. Thus, reproduction rates in any habitat depend on rainfall. Ultimately, too little rainfall translates into very limited aquatic habitat whereas too much can result in eggs and immature stages being washed away by water run-offs [39].

### 5.2. Changes in Ecosystems and Wildlife Habitats

Climate change is a significant factor influencing biodiversity and considerably impacts the structure of ecological communities [40]. Recent fluctuations in temperature, precipitation patterns, and environmental dynamics result in significant changes in the interactions among animal species, their habitats, and human populations. These evolving relationships create a complex array of elements that impact the transmission and prevalence of zoonotic diseases. There are implications for the survival and spread of zoonotic pathogens and their vectors’ reproductive rates and geographic distribution. Additionally, climate change is expected to influence activities that prolong the transmission of zoonotic diseases and enhance human exposure to the environment and, consequently, to vectors and their associated pathogens [41]. A primary ecological component of any zoonosis is the habitat that the reservoir host(s) occupies [42].

Climate change alters species richness in a specific geographical location by influencing the geographic distributions of species. Most mammal species are expected to encounter at least one unfamiliar species within their potential future range, regardless of the emissions scenario [43]. This impact occurs through modifying ecological habitat suitability, which can result in species’ decline in certain areas while enabling their expansion in others [44]. These shifts influence the spatial distribution of zoonotic diseases by introducing their hosts to new environments. Additionally, these changes can alter species composition and ecological dynamics, potentially resulting in new host–pathogen interactions. Such interactions may create novel transmission pathways that increase infectivity and contribute to the emergence of more harmful disease variants [43].

Climate-induced changes in ecosystems and habitats have resulted in shifts in wildlife distribution and migration patterns, bringing animals and humans closer and increasing the potential for zoonotic transmission [45]. This expansion could heighten the potential for increased interactions between different species. Consequently, these factors may elevate the risk of diseases that are either currently prevalent or documented in various world regions [46]. The spread and outbreak of zoonoses due to climatic changes can be precisely outlined into four stages. Firstly, changes in climate trigger animal migration from their natural habitats, including large animal herds and vectors of several diseases, thereby creating new connections between the wild environment and human-inhabited environments. Secondly, pathogen spillover is a systematic connection between global environmental changes and the emergence of diseases, eventually resulting in human infections. Thirdly, the infection can be transmitted within the human population, and the fourth stage is the exponential spread of the disease [47].

Changes in land use, deforestation, and urbanization can increase the proximity between humans and wildlife, heightening the risk of disease spillover. Consequently, diseases such as Ebola, Marburg, and the Nipah virus may shift from wildlife to humans due to these interactions [48]. The changing climate facilitates the transmission of diseases from animals to humans. Long-term shifts in temperatures and weather patterns can extend the geographic range of disease vectors, such as mosquitoes carrying diseases like Zika and malaria, to new areas previously unaffected. Furthermore, changes in precipitation can also affect the breeding habitats of disease-carrying animals, thereby affecting disease dynamics [49].

Climate warming impacts the availability of suitable habitats, mainly those necessary as refugia for ticks, hosts at appropriate densities, and mechanisms for dispersion (including wind and air currents for dipteran vectors and avian or mammalian hosts for ticks). Although the extent of its impacts will differ from one vector-borne disease to another, the overall effects of a warming climate on a changing geographic distribution may be the same for both ticks and dipteran vectors. What is important to note is that a warming climate will likely change the seasonality of vector activity. Earlier springs are expected to advance the seasonal activity of vectors in areas where they are already endemic, as has been observed for *I. scapularis* ticks in the northeastern USA [50]. The extreme temperature increase associated with climate change, drier climates, droughts, and severe floods will negatively influence the distribution, population density, egg production, developmental cycle, and over-winter ticks’ survival rate [8].

In recent years, as heatwaves and flooding have become more common in Europe and summers have grown longer and warmer, *Aedes albopictus* has established itself further north and west (ECDC). One challenge to maintaining progress in the fight against malaria in Africa is the expansion of vector populations into new regions [51]. The spread of dipteran vectors and their pathogens is likely to be more rapid and epidemic in nature compared to the spread of ticks and tick-borne pathogens. This is because dipterans are more mobile (ticks need to be dispersed by their hosts) and dipteran populations can become established quickly due to their short life cycles and immediate responsiveness to higher temperatures, whereas for tick populations, it may take years to adapt. This is primarily due to the outcome of a warmer climate on the length of their life cycle. Pathogen transmission by ticks relies on their multi-year life cycle, while dipteran-vectored transmission cycles can occur in weeks. The hesitancy to implement strict measures to curb several factors that impact the climate directly contributes to changing climate conditions, which favor the expansion of disease-carrying vectors to the most vulnerable communities already on the front lines of climate change.

### 5.3. Human Behavior and Exposure Risks

Human behavior, such as migration and urbanization, is characterized by rapid intensification of agricultural activities, socioeconomic change, and ecological breakdown. These can significantly impact the epidemiology of infectious diseases, resulting in habitat adjustment and changes in species distribution and contact rates that promote the emergence of zoonotic diseases [52]. An increase in agricultural activities that follow urbanization distorts, erases, and fragments previously intact ecosystems, creating habitats that bring humans and wildlife nearby. The reduction in spatially and phenologically diverse habitats affects the distribution patterns of reservoir hosts of zoonotic diseases over time and space, resulting in a more significant overlap with other vertebrate hosts, vectors, and human populations [53].

Migration, trade, sanitation, population density, access to clean water, and other human factors can increase the transmission rate of pathogens and alter vector dynamics. In contrast, social factors, socioeconomic status, housing, race, ethnicity, gender, and education influence health inequality and impact the epidemiology of infectious diseases in urban areas [54]. Recent research reveals that the loss of functional diversity due to non-random patterns of defaunation has significantly affected the zoonotic spillover risk. This is through the sparse distribution or invasion of opportunistic zoonotic hosts that flourish in human-modified landscapes or through the arrays of human-induced excision of predators and competitors of zoonotic species [53,55]. The commercial wildlife trade, introducing invasive species, and transporting livestock and companion animals enhance interaction diversity, create opportunities for cross-species transmission, and facilitate the emergence of new pathogens with zoonotic spillover.

## 6. Triple Planetary Crisis: Climate Change, Biodiversity Loss, and Pollution Roles in Zoonotic Disease Emergence

**Figure 6:**
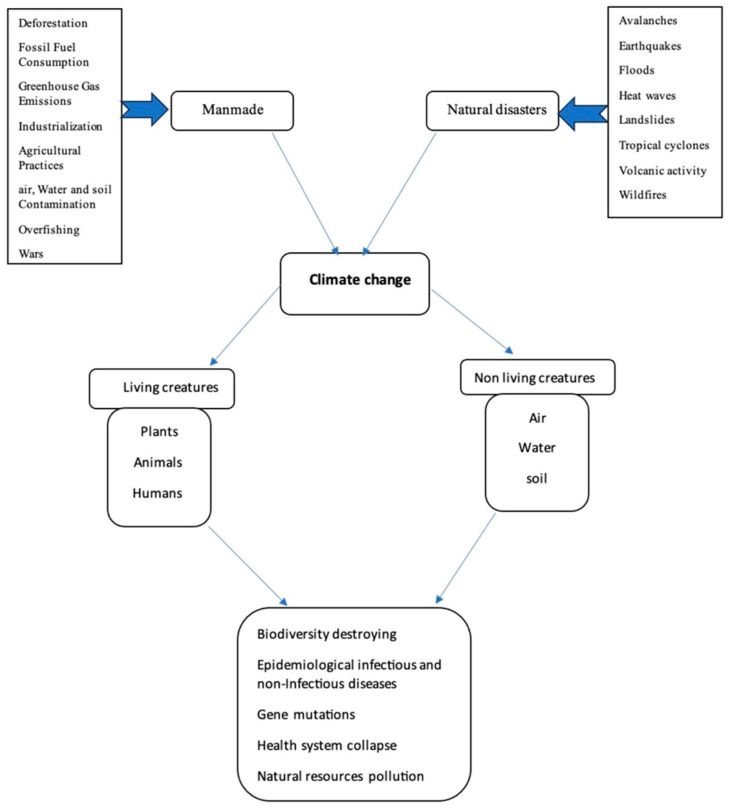
An abstract showing the implications of climate change on the ecosystem.

The triple planetary crisis is a new concept that was declared by the United Nations (UN) to drive world action effectively to save the future of Earth. Climate change, pollution, and biodiversity loss are three essential bodies of the Triple Planetary Crisis term [56]. Globally, UN initiatives encourage people to reduce their environment-harming activities and improve the quality of the ecosystem; however, deleterious human activities are still the predominant factor for climate change. Epidemiological studies have concluded that human-made activities, which are physical, chemical, and biological interactions, are increasingly shifting public health patterns into disease-dominating ones [57]. In addition, climate change catalyzes enormous consequences on human beings and the ecosystem through the imbalance it invokes in the components of biodiversity, as shown in Figure 6. These changes disturb the biodiversity system and increase the possibility of lethal attacks governed by microorganisms toward other living organisms, causing various diseases in humans and animals.

### 6.1. Pollution-Induced Immune Suppression and Increased Susceptibility to Infections

Air pollution is an interactive medium of climate change and health stability. This specific type of pollution causes severe consequences to humans due to inhaling air that contains pollutants of varying types. Air pollution includes indoor and outdoor contaminated air particles, which could be physical, chemical, and biological substances floating at the atmospheric level. Accordingly, greenhouse emissions produce harmful gasses such as carbon dioxide (CO_2_), methane (CH_4_), and nitrous oxide (N_2_O). These compounds are the main causes of the severity of air pollution in the environmental ecosystem [58]. Seven million premature deaths are estimated to be caused by air pollution annually, yet the rate is still increasing. Air pollution influences changes in the global ecosystem, especially air quality. Multiple chemical pollutants in breathable air spread globally, such as particulate matter (PM), infectious agents, and gas emissions [59,60,61]. The human immune system is sensitive to foreign bodies invading the respiratory tract. Immune cells produce chemicals and allergic substances to fight these invaders and protect their body systems. Consequently, the excessive stimulation of the immune system may accelerate the pathogenicity of infectious diseases and induce immunosuppression. Currently, particulate matter (PM2.5) is an integral component of air pollution that increases the provocation of the immune system in various respiratory diseases. Previous studies have shown that the diameter of different kinds of PM can stimulate multiple immune responses that further trigger the immune system. It is demonstrable that coarse PMs that are ≤10 μm (PM10) mostly accumulate in the upper airway, fine PMs that are ≤2.5 are in the central and peripheral airways and alveoli, and PMs with ≤0.1 μm Ultrafine (UFPs) deposit in the tissues of the respiratory tract [62,63].

Recent studies on human gene expression have indicated that biological biomarkers can distinguish and connect air pollution with the immune cell response to infection development. Marín-Palma’s research has found that human antiviral immune responses to particulate matter (PM10) have elevated proinflammatory cytokines, such as IL-1β and IL-6. At the same time, Van’s research established a link between the progression of diseases and cardiopulmonary responses to air pollution exposure. Additionally, Jung’s findings have highlighted the toxicological impact of cytokines and histone biomarkers, showcasing the interactions between particulate matter (PM2.5) and inflammation during pregnancy and its effects on newborns [64]. Conversely, Tripathy suggests prolonged exposure to air pollution components may trigger immune cell activation over the long term. In addition, the study recommends further research into long-term effects to better understand the primary relationships between exposure and inflammation [65].

### 6.2. Climate Effects on Human and Animal Health

Humans use pesticides to alleviate the harmful impacts of insects or plants, increasing agricultural production. In contrast to their temporary benefits, pesticides harm the ecosystem’s core components badly [66]. Some bacteria and fungi tolerate pesticide toxicity and metabolize these chemicals to be less toxic. Biodegrading microorganisms are microbes that minimize the effect of pesticides in sewage and ecosystems by metabolizing pollutants and complex chemical substances. However, humans and animals cannot metabolize toxic substances accumulated in their bodies as they lack the specialized enzymes needed to minimize the toxicity of these pesticides [66,67,68]. Farmers who use insecticides that contain organophosphates are exposed to develop gene mutations rapidly. In parallel, people who live or consume agricultural products where insecticides or pesticides are applied are prone to developing chronic diseases and gene disorders [69,70,71]. DNA methylation, which is caused by exposure to pesticides, has deleterious impacts on human genes. The intoxications of Organophosphate pesticides cause polymorphism due to their ability to enhance oxidative stress that impairs DNA functions, including protein synthesis. Embryos are susceptible to advanced gene mutations in both humans and animals. Endocrine disruptors (ED) can cause gene polymorphisms, leading to various metabolic and endocrine diseases. Pesticides cause an imbalance in human cells by interacting with hormonal receptors and impairing gene expression and cell growth, leading to mutations, decreased infertility rate, immunosuppression, and cancers [72,73,74,75].

Organophosphates induce environmental harm to aquatic creatures, especially fish, when they reach their internal organs and bioaccumulate. It may cause a malfunction in the immune system, cause a gene mutation, and induce microbial disease growth. Evidence has claimed that a fragile ecosystem takes a long time to bring about change and improvement. In addition, climate change is a multifactor determinant that influences chemicals to deteriorate the biodiversity of the global ecosystem and increase the mortality risk for all living creatures [76,77,78,79,80]. Fresh water is a medium for plenty of biological interactions in the ecosystem. Manufacturing activities and natural disasters simultaneously affect fresh water by increasing the burden of various diseases. Microplastics (MPs) are made that are measured less than 5 mm. Humans have voluntarily introduced pesticides and microplastics to eradicate harmful microorganisms through occupation or food acquisition activities. Marine biodiversity is severely affected by these noxious chemical substances cast into the aquatic environment. Fresh water is a natural resource for humans, animals, and plants; however, multiple biohazardous chemicals have put these natural resources at risk. Naturally, microorganisms are essential in keeping the ecosystem safe and sustainable. MPs are still posing an environmental challenge that has driven the attention of many climate scientists to investigate this situation extensively. The outcomes of unneeded plastic production or fossil fuel are turned into viable sources of MPs, and there is a possible chance that these chemicals could damage the whole marine system besides plants, animals, and humans [81,82,83,84].

Humans and animals suffer from the climate crisis, and non-living things undergo drastic natural changes. Soil, water, air, and green space are natural resources that show ecological robustness in the face of environmental destruction. In the past few years, big cities have gradually established national laws of renewable and nonrenewable resource demands to moderate the impact of climate emergency. Many international initiatives encourage people to decrease their electricity use, cars, and chemicals to reduce the possible changes in the ecosystem. Fresh-water and food contamination, waste management, and pollution are three bold factors that severely accelerate the climate conditions in urban and industrialized areas [85]. Socioeconomic factors impact the emergence of infectious diseases following global climate crises in both urban and non-urban areas. Notably, people in urban areas are much more likely to develop infectious disease complications than those in industrial areas. If tropical climate changes, pandemic diseases, and limited access to healthcare services in metropolitan areas continuously rise, more robust health systems will be needed to tolerate the recent global trends [86]. Hence, climate change impacts the prospective estimation of vector-borne diseases to increase sharply worldwide. Malaria and dengue are vector-borne diseases that threaten health security in many countries due to global warming. Conversely, interesting studies on climate change revealed that vector-borne diseases may interchangeably increase or decrease among humans and animals according to the climate crisis, demographic factors, socioeconomic status, and response rate of the health system in urban and non-urban areas [87,88,89,90,91].

### 6.3. Host–Pathogen–Environment Interactions and the Role of Pollutants in Zoonotic Diseases

Biologically, it is notable that heavy metals impact the living creatures in the environment. Heavy metals negatively change the agroecosystem as long as humans consume mainly plant- and animal-based diets. Heavy metals affect plants’ nutrition and break down the essential biochemical interactions in soil, air, and water components that plants need to fulfill their metabolic needs [92]. Heavy metals are the elements found in living organic cells with no or little benefit and need a very long time to be metabolized in living organs. The bioaccumulation and long half-lives of heavy metals could exert exertion on the human immune system. These processes can also cause cell malfunction and ease the penetration of microorganisms into human cells, leading to high infection rates [93]. Heavy metals exist in the environment with low concentrations; however, once they reach the organs or cells, they bioaccumulate and alter the standard functions in the host. How heavy metals disrupt the environment could lead to a steep decline in biodiversity. Ultimately, the inconsistency of the ecosystem catalyzes the deterioration of the function of natural microbiota, which can lead to superinfecting human and animal cells due to their altered immune systems [94]. Human microbiota interacts positively with certain trace elements to perform the metabolism process efficiently, build up proteins, and detoxify exogenous compounds or administered drugs.

In contrast, due to climate change, heavy metals have been overexposed in the air, water, and food. Trace metals can damage these chemical interactions and change the function of microbiota components, severely impacting the immune system [95,96,97]. Cadmium is the most common element of heavy metals that threatens the lives of humans and animals. Predominantly, manufacturing activities produce cadmium elements inhaled easily by the human respiratory system or accumulate in the soil, which plants grow and absorb accordingly. In addition, Cadmium triggers the immune cells, such as macrophages, and impairs their ability to devour pathogens [98]. The rising mercury toxicity has heavily directed the scientific community toward studying its impacts on humans and animals. Scientifically, mercury traces produced from industrial sources, such as Elemental mercury (Hg 0), intimidate living organisms because it is insoluble in water and highly volatile [99]. Typically, many products incorporate mercury for technological use, such as thermometers and blood pressure tests in the medical field. Unfortunately, Hg 0 deposits in the respiratory tract cells that humans cannot easily be removed. The higher the extent of mercury bioaccumulation, the more health problems it could lead to, such as neurological disorders [100,101,102]. Moreover, Mercury affects the programmed cell death in lymphocytes and influences microorganisms to grow smoothly. However, Yang revealed that further studies on heavy metals are needed to link them with the omics of living organisms, mainly humans, unfolding the root effects on human genes and deterioration to the environmental components [103].

## 7. Mitigation and Adaptation Strategies from the One Health Approach Prospective

### 7.1. Strengthening Surveillance and Early Warning Systems

#### 7.1.1. Definition and Purpose of the Integrated One Health Surveillance

Surveillance is a critical component of the One Health approach in the context of zoonotic diseases and climate change. One Health surveillance integrates data concerning humans, animals, and the environment, supporting informed decision-making for effective health interventions grounded in evidence and systems thinking [104,105]. One Health surveillance involves systematically collecting, validating, analyzing, interpreting, and disseminating data concerning humans, animals, and the environment. This holistic approach ensures continuous awareness of population disease trends, enabling timely interventions to reduce morbidity, mortality, and economic losses [106]. The primary aims of zoonotic disease surveillance include the early detection of exotic, emerging, and re-emerging diseases, demonstrating freedom from disease, and monitoring endemic conditions [105,106]. There are multiple surveillance types to encompass active, passive, and sentinel approaches, extending beyond infectious diseases to include various health determinants, such as risk behaviors and environmental health factors [107]. Effective surveillance systems must detect health events, consolidate relevant data, confirm outbreaks, analyze trends, and provide feedback to data sources to inform ongoing public health strategies [108]. The Effectiveness of the One Health surveillance can be significantly enhanced by broadening its focus to incorporate social and economic factors alongside behavioral risk assessments. International surveillance programs such as GLEWS (Global Early Warning System for Major Animal Diseases and Zoonoses) and GOARN (Global Outbreak Alert and Response Network) play a pivotal role. These programs have already made significant strides in addressing disease transmission by fostering international collaboration, enhancing the capacity for rapid response to outbreaks, and ensuring that countries have the tools and frameworks to manage health crises effectively [109,110]. Investing in robust surveillance systems is essential for ensuring a rapid response mechanism that can adapt to the dynamic nature of zoonotic diseases, particularly in light of climate change and increased human–animal interactions.

#### 7.1.2. Case Studies Demonstrating the Integrated One-Health Surveillance

Example 1: The PREDICT Project by USAID

The PREDICT Project serves as a model for global surveillance, successfully identifying over 1000 viruses in wildlife across 36 countries to enhance preparedness against zoonotic diseases. The primary goal of the PREDICT Project is to shift countries from a reactive post-outbreak response to a more proactive approach to managing zoonotic diseases. For instance, in the Democratic Republic of Congo (DRC), the project employed a One Health approach to respond rapidly to an Ebola virus disease outbreak, which led to swift containment efforts [111].

Example 2: Participatory One Health Disease Detection (PODD) System in Thailand

The PODD system is a community-owned surveillance initiative designed to detect and control disease outbreaks early. This mobile application empowers local volunteers to report health events directly through smartphones, facilitating rapid identification of potential outbreaks. Over a 16-month pilot, the app recorded 1029 true reports of abnormal health events in real time, demonstrating its effectiveness in outbreak management [16,112].

Example 3: Cambodia National Health Hotline

In January 2016, Cambodia launched the National Health Hotline to improve disease reporting and response, providing a vital resource for the public and healthcare workers. The hotline enabled efficient communication regarding health concerns, especially in remote areas [113,114]. Additionally, the country employed mathematical models to analyze animal movement practices in southern Cambodia, which helped assess the potential spread of H5N1 from live bird markets [115]. Later, after COVID-19, one study highlighted that the hotline was one of the reasons for Cambodia’s early success in responding to COVID-19. By facilitating efficient disease reporting and leveraging international support, Cambodia demonstrated the critical importance of integrated health communication systems to manage public health crises [115].

Example 4: AfyaData: Advancing Disease Surveillance in Tanzania.

The AfyaData application represents a significant advancement in African One Health disease surveillance. This mobile platform is another example of how community health reporters can be equipped to collect and submit real-time data on disease occurrences. Within five months of its launch, AfyaData documented 1915 clinical cases, illustrating its capacity to enhance local health monitoring [116]. AfyaData has also proven its utility during the recent Ebola outbreak in the Democratic Republic of Congo, where it was utilized for syndromic surveillance [110]. This innovative approach facilitates timely reporting and strengthens community engagement in disease detection and response efforts.

#### 7.1.3. Utilizing Evaluation Tools

Creating an effective surveillance system for infectious diseases is complex, particularly in resource-limited countries, where poor data integration, fragmented data streams, and limited private sector involvement hinder public health responses. Vital registration systems often operate independently of healthcare systems, leading to underreporting of deaths and inadequate understanding of disease patterns. Additionally, separate healthcare utilization and outbreak reporting create inconsistencies, while decentralized recruitment processes contribute to human resource issues [117]. The OH-EpiCap tool is a comprehensive evaluation aid designed to assess and enhance the capacities and capabilities of One Health surveillance systems. It offers a structured solution whereby these systems are evaluated across three dimensions: organization, operation, and impact. OH-EpiCap facilitates collaboration among sectors through a standardized scoring system, which enables stakeholders to identify strengths and weaknesses, improve data quality, and enhance responsiveness to emerging health threats. By addressing these challenges, OH-EpiCap plays a crucial role in strengthening public health infrastructure and ensuring timely and effective responses to infectious disease outbreaks [117,118].

### 7.2. Enhancing Interdisciplinary Collaboration

The One Health approach emphasizes collaboration across various sectors, including public health, veterinary, environmental, and others [104]. Improving interdisciplinary collaboration is crucial in tackling the complex interactions that drive the emergence of zoonotic diseases and the impacts of climate change. A study examining Tick-Borne Diseases (TBD) surveillance systems in the Netherlands, Spain, and Italy provides a compelling case for this approach. The study used a One Health evaluation protocol to identify differences among the three surveillance systems. In the Netherlands, the surveillance system demonstrated high transdisciplinary collaboration, with suitable identification of the actors and public engagement in research and education. This collaborative approach led to measurable outcomes such as reducing tick bites and discovering new pathogens and tick species. In contrast, the surveillance systems in Italy and Spain, which are based on compulsory notification to health authorities, showed less effectiveness. The enforcement of legislation and the availability of economic resources were fragmented and limited to the most severe diseases. The non-scientific community was marginally considered, and collaborations were limited to local initiatives [118]. This study underscores the effectiveness of interdisciplinary collaborations in disease prevention and early response to emerging health threats, including TBD. It also highlights the importance of adopting an integrated approach for zoonosis surveillance and management, as advocated by the One Health approach. In Peru, integrating Earth observation (EO) technologies exemplifies how improved interdisciplinary collaboration can enhance health outcomes. Researchers can monitor environmental changes contributing to zoonotic diseases’ emergence by utilizing satellite imagery and remote sensing data. For instance, tracking deforestation and land-use changes provides insights into how these factors influence the habitats of disease vectors, such as ticks and mosquitoes. Collaborations between environmental scientists, public health officials, and local communities facilitate the timely identification of potential health risks in this hotspot for dengue fever and leishmaniasis, driven by its tropical Amazonian climate [106]. This approach not only aids in predicting and preventing disease outbreaks but also enables effective public health interventions tailored to the local context. Incorporating Earth observation data into the One Health framework highlights the power of interdisciplinary partnerships in addressing complex health challenges, ultimately fostering a more resilient response to the evolving threats posed by zoonotic diseases in a changing climate [107].

### 7.3. One Health Zoonotic Disease Prioritization (OHZDP)

Defining and prioritizing the highest health risks is crucial and essential for better utilization of available resources in addition to capacities, advanced technology, and research, as well as developing strategies and data-driven policy recommendations for better prevention, preparedness, detection, and response measures. As an integrated One Health approach is substantial for progress in combating health threats, priorities across related sectors and stakeholders should be harmonized to sustainably balance and optimize the health of humans, animals, and ecosystems. The OHZDP process is a comprehensive multisectoral approach to identify and prioritize zoonotic diseases of most significant concern, which burden the public health sector. By fostering collaboration among One Health partners, OHZDP determines priority diseases and gives insights into the following steps and action plans to address them. The OHZDP process brings together representatives from human, animal, and environmental health sectors, besides other relevant partners, to prioritize zoonotic diseases of most significant concern for multisectoral One Health collaboration in a country, region, or other area. This process uses a transparent approach and incorporates equal input from all represented One Health sectors working at the human–animal–environment interface [119].

#### 7.3.1. Goals of the OHZDP Process

The process is obtained and achieved to prioritize zoonotic diseases of most significant concern affecting the public health in each country, besides developing and giving insights for the next steps and action plans to address those priority zoonotic diseases in collaboration with One Health partners.

#### 7.3.2. Expected Outcomes of the OHZDP Process

The process obtains a list of the priority zoonotic diseases with the highest concern, as agreed upon by all represented One Health sectors. Afterward, it presents recommendations for the next steps, operational plans, and action plans for multisectoral One Health engagement to address the priority zoonotic diseases. Furthermore, it gives insights for understanding and defining the roles and responsibilities of all represented One Health sectors, which will, in turn, strengthen multisectoral One Health coordination mechanisms and networks. Eventually, it generates a report highlighting the outcomes of the approach to help advocate for One Health priorities [119].

#### 7.3.3. Benefits of the OHZDP Process

The OHZDP process strengthens the overall connection among representatives across the human, animal, and environmental health sectors and other relevant partners for building a foundation of trust through multisectoral One Health collaboration, coordination, and communication. Also, the prioritization of zoonotic diseases would use equal input from all represented One Health sectors through a transparent and collaborative process [120]. Moreover, it supports the creation of multi-sectoral communication through One Health coordination mechanisms. This allows for effective coordination and targeting of limited resources to effectively build capacity and collaboration in a way that prioritizes zoonotic diseases [121]. It also implements adaptability approaches that consider the local context, reflecting voting members’ criteria relevant to their country or region. It would provide scalable data collection applicable across subnational, national, and regional levels and can be adapted to other infectious diseases and One Health issues. Consequently, it informs assessments, planning efforts, or strategy development relevant to the One Health approach. Eventually, it generates real-time outcomes and reports, showing an updated list of priority zoonotic diseases, next steps, action plans, and formal reporting mechanisms [119].

## 8. Future Considerations and Research Gaps

### 8.1. Identified Research Gaps

Scientific research needs to continue to trail the root causes of climate change. To date, GHG’s major and minor influences could accelerate pollution dramatically at the atmospheric level. What is more, researchers need to include the role of genomic and genetic variation impacts on climate equilibrium in humans and other living creatures and disclose the most unpredictable factors to save the diversity of the biosphere. Humans can no longer overcome the consequences of global warming when there are no strong effective policies to cut off the misbehavior of harmful chemicals that are used carelessly. Several studies have been carried out to understand the impacts of climate change on ecosystems and the transmission of zoonotic diseases. However, there are still pending research gaps that need to be addressed. One crucial area that requires critical investigation is the relationship between climate-driven wildlife migration, habitat shift, and change in vector ecology and their effects on host–pathogen interactions. Although some species migration has been documented, a more in-depth analysis is needed to comprehend how these ecological changes can modify disease dynamics and establish new transmission pathways. Finally, there are numerous opportunities for interdisciplinary studies that integrate climate science, ecology, public health, and social sciences. Such studies should aim to create predictive and futuristic models that can inform global policies and enhance preparedness for zoonotic diseases associated with climate change. Fundamentally, we emphasize the need for future research to identify gaps and address existing ones embedded in current practices and policies. Focusing on innovative strategies that reinforce sustainability can primarily enhance our capacity to prevent and manage zoonotic threats.

### 8.2. Proposed Future Considerations

Significant barriers, particularly in developing countries, hinder the implementation of One Health strategies. These challenges include weak governance, poor collaboration, inadequate planning, and limited resources. Overcoming these obstacles requires coordinated efforts at both national and international levels, emphasizing the improvement in governance, enhancing surveillance, and fostering community engagement. By addressing these barriers, the One Health approach can better mitigate future pandemic risks and promote sustainable health outcomes for humans, animals, and the environment. Additionally, aligning actions to combat climate change with disease prevention efforts strengthens global health security. Another consideration should be the long-term aftermath of habitat fragmentation on zoonotic diseases. Climate change, while impacting ecosystems, promotes the survival and persistence of vectors and pathogens, particularly in urban environments with contact between humans and wildlife. Understanding how these dynamics influence vector populations and migration is essential for developing effective disease prevention strategies. Promoting sustainable practices and policies is increasingly critical in addressing zoonotic diseases, especially within the One Health framework. To effectively advance these sustainable initiatives, aligning our policies with the United Nations Sustainable Development Goals (SDGs) is imperative. By doing so, we establish a global alliance that adopts strategic frameworks, ensuring the interventions are immediate and sustainable in the long run. Aligning the efforts with the SDGs recognizes the importance of including global or international actors to pragmatically tackle the complex interrelationships between human health, animal health, and environmental integrity. This holistic perspective is crucial in reducing the risk factors associated with zoonotic diseases, which are interlinked and often exacerbated by environmental degradation and climate humiliation.

## 9. Conclusions

Promoting sustainable practices and policies is increasingly recognized as vital in the fight against zoonotic diseases, particularly in One Health. The fruits of multi-sectoral and multidisciplinary collaboration have been gained through processes like OHZDP and approaches like One Health, among other theories, panels, and frameworks, all of which are trusted as strides in the prevention and protection against vicious health events threatening all lives on earth. The interplay between the One Health approach, climate change, and zoonotic diseases is essential for understanding how global environmental changes influence the emergence and the spread of these diseases. The One Health framework offers a holistic strategy to address global health challenges by integrating human, animal, and environmental health. That said, it is critical to note that etiologies, ways of transmission, and reservoir–host variability are unsteadily altering as fast as climate and environment are mutating. With that in mind, pathogenic microorganisms are continuously in the loop of mutation and evolution, consequently necessitating steady and up-to-date monitoring of emergent zoonoses. The intricate interconnectedness and interactions between humans and other forms of life generate even more complex zoonoses that obligate the strengthening of our defensive mechanistic adaptation and response through research, collaboration, policymaking, and governance. Recent pandemics demonstrated that health threats are complex and interconnected, exacerbated by socio-economic, environmental, and political factors. To address these challenges, key global organizations developed the One Health Joint Plan of Action (OHJPA) to tackle health issues arising from the interaction between humans, animals, plants, and the environment. Early detection, effective preparedness, and prevention measures could facilitate better control over emergent zoonoses. A proactive and integrated approach will build resilience against potential outbreaks and ensure a healthier future for our planet and its inhabitants.

## Abbreviations

CH_4_	Methane
CO_2_	Carbon dioxide
DNA	Deoxyribonucleic acid
DRC	Democratic Republic of Congo
ED	Endocrine Disruptors
EID	Emerging Infectious Diseases
ENSO	El Niño-Southern Oscillation
EO	Earth Observation
FAO	Food and Agriculture Organization
GHG	Greenhouse Gases
GLEWS	Global Early Warning System for Major Animal Diseases and Zoonoses
GOARN	Global Outbreak Alert and Response Network
MPs	Microplastics
N_2_O	Nitrous Oxide
OH	One Health
OHJPA	One Health Joint Plan of Action
OHHLEP	One Health High-Level Expert Panel
OHZDP	One Health Zoonotic Disease Prioritization
PODD	Participatory One Health Disease Detection
PM	Particulate Matter
SDGs	Sustainable Development Goals
TBD	Tick-Borne Diseases
ToC	Theory of Change
UFPs	Ultrafine Particles
UN	United Nations
UNEP	United Nations Environment Program
WHO	World Health Organization
WOAH	World Organization for Animal Health
